# Pharmacokinetics, Tissue Distribution, Metabolism, and Excretion of Naringin in Aged Rats

**DOI:** 10.3389/fphar.2019.00034

**Published:** 2019-01-28

**Authors:** Xuan Zeng, Weiwei Su, Yuying Zheng, Yudong He, Yan He, Hongyu Rao, Wei Peng, Hongliang Yao

**Affiliations:** ^1^Guangdong Engineering and Technology Research Center for Quality and Efficacy Reevaluation of Post-Market Traditional Chinese Medicine, Guangdong Key Laboratory of Plant Resources, School of Life Sciences, Sun Yat-sen University, Guangzhou, China

**Keywords:** naringin, naringenin, ADME, aged rats, LC-MS/MS

## Abstract

Aging is an inevitable biological process characterized by the loss of functional capacity and associated with changes in all phases of pharmacokinetic processes. Naringin, a dietary flavanone glycoside, has been proved to be beneficial for the treatment of multiple age-associated chronic diseases. To date, the pharmacokinetic processes of naringin in aged individuals are still unknown. Thus, a rapid resolution liquid chromatography tandem triple quadrupole mass spectrometry (RRLC-QQQ-MS/MS) method was established for the determination of naringin and its metabolite naringenin in rat plasma, urine, feces, and tissue homogenate. The pharmacokinetic parameters were calculated and a higher exposure of naringin and naringenin were observed in aged rats. Naringin and naringenin were mostly distributed in gastrointestinal tract, liver, kidney, lung, and trachea. Furthermore, a total of 39 flavonoid metabolites (mainly glucuronides and sulfates) and 46 microbial-derived phenolic catabolites were screened with ultra-fast liquid chromatography-quadrupole-time-of-flight tandem mass spectrometry (UFLC-Q-TOF-MS/MS). Naringenin, hippuric acid, and 3-(4’-hydroxyphenyl)propionic acid were predominated metabolites. This study systemically investigated the pharmacokinetics, tissue distribution, metabolism, and excretion of naringin in aged rats, revealing age- and gender-related changes in the *in vivo* behavior of naringin. These results would be helpful for the interpretation of pharmacokinetics and pharmacodynamics of naringin in aged population.

## Introduction

Aging is an inevitable biological process characterized by the loss of functional capacity of several organs as well as to the reduced efficacy of homeostatic mechanisms, and consequently associated with changes in all phases of pharmacokinetic processes, including absorption, distribution, metabolism, and excretion (ADME) (Corsonello et al., 2010). Recently, population aging is progressing rapidly in many countries (Paré et al., 2007). Aged people generally suffer from multiple chronic diseases and take multiple medications (Kaufman et al., 2002). Unfortunately, elderly patients are frequently excluded from clinical trials and as a consequence achieved scientific evidences and indications may be of limited usefulness in aged population (Spall et al., 2007).

Naringin, chemically known as 5,7,4′-trihydroxyflavanone-7-*O*-rhamnoglucoside, is a common dietary flavanone glycoside derived from citrus fruits. As a typical example for the term “phytopharmaceutical,” naringin has been experimentally documented to possess potent pharmacological activities and therapeutic potential for the treatment of multiple illnesses, including inflammation (Ahmad et al., 2015), diabetes (Punithavathi et al., 2008), cardiovascular disorders (Alam et al., 2013), metabolic syndrome (Alam et al., 2014), neurodegeneration (Gopinath and Sudhandiran, 2016), and respiratory diseases (Lin et al., 2008; Gao et al., 2011; Shi et al., 2017), which are mostly age-associated chronic diseases (Akushevich et al., 2013; Wang et al., 2018). Recently, naringin has been approved for clinical trials by China Food and Drug Administration (Li et al., 2015). Given the healthy benefits, naringin-containing nutraceuticals are increasingly used as dietary supplements (Lin et al., 2014), especially in aged population. However, to date, the pharmacokinetic processes of naringin in old people have not been well investigated, as well as in aged animals.

Profiling of pharmacokinetic properties is vital to the understanding of the *in vivo* behavior and action mechanism. Thanks to the diverse bioactivities, naringin has been involved in many metabolism and excretion studies performed in adult individuals, including mice (Naiara et al., 2015), rats (Liu et al., 2012), rabbits (Hsiu et al., 2002), dogs (Matabilbao et al., 2007), and human (Zeng et al., 2017a). Generally, when orally administrated, naringin is hydrolyzed to its aglycon naringenin by lactase-phlorizin hydrolase and intestinal microflora (Chen et al., 2014). Naringenin is partly absorbed and then engaged in both phase I and phase II metabolism (Zeng et al., 2018b). Meanwhile, unabsorbed naringenin and the metabolites excreted by the enterohepatic circulation are further degraded into phenolic catabolites by intestinal microbiota (Roowi et al., 2009; Pereira-Caro et al., 2016). As ADME processes are associated with age, it is meaningful to evaluate the effects of age-related changes on the *in vivo* biotransformation of naringin.

In the current study, we systemically investigated the pharmacokinetics, tissue distribution, metabolism, and excretion of naringin in aged rats after a single oral administration of naringin. The pharmacokinetic parameters and tissue distribution for naringin and its metabolite naringenin, which possesses similar biological activities (Rani et al., 2016), were determined. Metabolites were screened in the urine and feces collected post dose and the metabolism pathways were proposed. Furthermore, the excretion properties of naringin and its main metabolites were elucidated. Obtained results would be useful in the interpretation pharmacokinetics and pharmacodynamics of naringin in aged population.

## Materials and Methods

### Chemicals and Materials

The reference standards naringin (purity: 94.7%), apigenin (purity: 99.6%), and hippuric acid (purity: 99.9%) were obtained from the National Institute for the Control of Pharmaceutical and Biological Products (Beijing, China). Naringenin (purity: 99.5%), hesperetin (purity: 95.0%), 4-hydroxybenzonic acid (purity: 99.0%), 3-(4’-hydroxyphenyl)propionic acid (purity: 98.0%), β-glucuronidase/sulfatase (Type H-1), and mass spectrometry (MS) grade formic acid were acquired from Sigma-Aldrich (St. Louis, MO, United States). Eriodictyol (purity: 97.0%), 5,7-dihydroxychromone (purity: 95.0%), and phloroglucinol (purity: 98.0%) were purchased from Sinova (Shenzhen, China). Hesperetin-7-*O*-glucuronide (purity: 95.0%), hesperetin-3′-*O*-glucuronide (purity: 95.0%), and hesperetin-7-*O*-sulfate (purity: 95.0%) were obtained from Toronto Research Chemicals (Toronto, ON, Canada). Naringenin-7-*O*-glucuronide (purity: 97.0%) was purchased from Cayman Chemical Company (Ann Arbor, MI, United States), while naringenin-4′-*O*-glucuronide (purity: 96.0%) was acquired from Shanghai ZZBIO Co., Ltd. (Shanghai, China). And the stable isotope labeled internal standard [2′,3′,5′,6′-D_4_]-4,6,4′-trihydroxydihydroaurone (purity: 94.5%) was supplied from Artis-chem Co. Ltd. (Shanghai, China).

Methanol of MS grade was purchased from Fisher Scientific Inc. (Fair Lawn, NJ, United States). Acetonitrile and ethyl acetate were obtained from Honeywell B&J Chemicals Inc. (NJ, United States). Deionized water was prepared by the Milli-Q system (Millipore Corporation, Billerica, MA, United States) and filtered through a 0.22 μm membrane filter before use. Naringin powder, for oral administration, was extracted from Exocarpium Citri Grandis with a purity of 98.8%.

### Animals

In this study, 20-month-old male (812 ± 69 g) and female (623 ± 73 g) Sprague-Dawley rats were obtained from Chengdu Dossy Experimental Animals Co. Ltd. (Chengdu, China, Certificate No. SCXK2015-030). The animals were acclimated for at least 1 week before the initiation of dosing with free access to food and water. Environmental conditions were maintained at 20-25°C, 55 ± 15% relative humidity, 12 h light/dark cycles and 12 air change cycles/h. The animals were fasted overnight with water available *ad libitum* before experiment. All experimental procedures and protocols complied with the National Institutes of Health guide for the care and use of Laboratory animals (NIH Publications No. 8023, revised 1978), and were approved by the Animal Ethics Committee of the School of Life Sciences in Sun Yat-sen University.

### Administration and Sampling

Early, we have investigated the absorption, tissue distribution, metabolism, and excretion of naringin in adult rats by oral administration in dose of 42 mg/kg (Liu et al., 2012; Zou et al., 2012). As the purpose of this work was to compare the ADME behavior of naringin in aged rats with that in adult rats, the same dose (42 mg/kg) and route of administration (gavage) were adopted.

Twelve aged rats (half male and half female) were randomly selected and administrated naringin by oral gavage (42 mg/kg). Blood samples (300 μL) were collected from retro orbital plexus into heparinized 1.5 mL polythene tubes before the administration and at 0.25, 0.5, 1, 2, 3, 4, 6, 8, 10, 12, 24, and 36 h post dose. Plasma samples were obtained by immediate centrifugation (2348 × *g* for 10 min).

In tissue distribution study, 96 rats (half male and half female) were randomly assigned into eight groups corresponding to the eight collection time points (0.25, 1, 3, 6, 8, 10, 15, and 24 h post dose) and each orally administrated with 42 mg/kg naringin. Blood samples were collected from retro orbital plexus and centrifuged to obtain plasma samples. After cervical dislocation, gastrointestinal tract (including stomach, duodenum, jejunum, ileum, and colon), liver, kidney, heart, lung, trachea, spleen, brain, fat, and skeletal muscle were removed from rats at designated time points. These tissues were washed with saline and dried with filter paper. The chyme in gastrointestinal tract was removed before the wash. For extraction, rat tissues were accurately weighed and then homogenized in saline (5 mL/1 g tissue, 20 mL/1 g for trachea) on ice with an IKA T 10 basic homogenizer (Staufen, Germany).

For metabolism and excretion study, 12 rats (half male and half female) were orally administrated with 42 mg/kg naringin and housed individually in metabolic cages (Y-3102, Yuyan Instruments Co. Ltd.; Shanghai, China), with fasting and free access to water. Urine and feces samples were collected before the administration and at time intervals of 0–4, 4–8, 8–12, 12–24, 24–36, and 36–48 h post dose. The volume of urine from each time interval were measured before cryopreservation. Feces samples were lyophilized, weighed, and then pulverized. The pulverized feces were extracted with saline (20 mL/1 g feces). After vortexed for 1 min, the mixtures were centrifuged at 2,348 × *g* for 1min, and obtained supernatant was applied for bioanalysis. Blank plasma, tissue, urine, and feces samples were obtained from rats without any drug administration and then used for the method development and validation. All obtained biological samples were stored at -70°C until analysis.

### Methods Validation and Quantitative Determination

The quantitative methods were developed based on reported studies (Fang et al., 2006; Liu et al., 2012; Zou et al., 2012; Zeng et al., 2017c, 2018a), and validated with reference to the Guidance for Bioanalytical Method Validation issued by Chinese Pharmacopoeia Commission in 2015. The specificity of quantitative method was evaluated by comparing chromatograms of the standard-spiked samples with the biological samples from six different sources. A calibration curve was composed of a blank sample, a zero-concentration sample (with internal standard) and eight concentration levels of samples covering the designated range, including lower limit of quantification (LLOQ). Calibration curves were constructed and fitted by linear least-squares regression analysis to plot the peak area ratio of analyte relative to the internal standard against the analyte concentrations. Precision and accuracy were assessed by analyses of repeated quality control (QC) samples (*n* = 6) at LLOQ and low, middle, high QC concentration levels in three separate runs for at least 2 days. Within-run and between-run precision were expressed by the relative standard deviation (RSD, %). Blank biological samples from six different sources were used to evaluate the matrix effect at low and high QC concentration levels. The matrix effect was calculated by comparing the peak area ratio of analyte relative to the internal standard in the analyte-spiked post-extracted sample with that acquired using a neat solution.

Rat plasma (50 μL) was mixed with 10 μL β-glucuronidase/sulfatase solution (10 unit/μL, dissolved in 0.2 mol/L acetic acid buffer, pH = 5.0) and incubated at 37°C for 2 h (Fang et al., 2006). After that, 10 μL internal standard spiking solution was added to the sample, followed by 500 μL ethyl acetate. After a vortex-shaking for 1 min and centrifugation at 10,000 × *g* for 10 min at 4°C, 450 μL organic layer was transferred into a fresh tube and then evaporated to dryness under a gentle stream of N_2_ at 37°C. The residue was reconstituted in 100 μL mobile phase. Samples were ultrasonic extracted for 3 min, vortex-mixed for 3 min, and centrifuged at 15,000 × *g* for 30 min at 25°C. Finally, an aliquot of 10 μL supernatant was injected for RRLC-QQQ-MS/MS analysis. Rat tissue homogenate (100 μL) was processed following the same procedures of plasma with two folds volume of working solutions (Zou et al., 2012). After extraction, the residue was also reconstituted with 100 μL of mobile phase, and 10 μL supernatant was applied for analysis. As to urine and feces homogenate, 100 μL liquid sample was mixed with 10 μL β-glucuronidase/sulfatase solution (20 unit/μL) and incubated at 37°C for 2 h (Liu et al., 2012). After adding 200 μL acetonitrile (dissolving stable isotope labeled internal standard), the sample was vortex-mixed for 3 min, centrifuged at 15,000 × *g* for 30 min at 25°C. Subsequently, 10 μL supernatant was subject to the analysis system.

For the determination of naringin and total naringenin in biological samples, a liquid chromatography-triple quadrupole mass spectrometry (LC-MS/MS) method was applied on Agilent 1200 RRLC tandem 6410 triple quadrupole mass spectrometers with an electrospray ionization source (ESI) (Agilent Technology, Santa Clara, CA, United States). Chromatographic separation was performed on an Agilent Poroshell 120 EC-C_18_ column (3.0 × 50 mm, 2.7 μm) tandem pre-column of Welch Analytical Guard Cartridges Ultimate XB-C_18_ (2.1 × 10 mm, 3 μm) at column temperature of 40°C. The mobile phase consists of 0.1% formic acid (*v/v*) in methanol (A) and 0.1% formic acid (*v/v*) aqueous solution (B). For plasma and tissue homogenate, the linear gradient elution program was: 0–1.6 min, 60–0% B; 1.6–2.0 min, 0% B; 2.0–2.1 min, 0–60% B; 2.1–4.0 min, 60% B. As urine and feces samples, to eliminate the severe matrix effect in excreta and determine other metabolites simultaneously, a different elution program was set as follows: 0–1.6 min, 80–0% B; 1.6–2.6 min, 0% B; 2.6–2.7 min, 0–80% B; 2.7–5.0 min, 80% B. The flow rate was 400 μL/min, and RRLC effluent was introduced directly to the mass spectrometer without splitting.

Mass spectrometry detector was operated in negative and multiple reaction monitoring (MRM) mode at unit mass resolution with a dwell time of 200 ms for all test compounds. The transitions and corresponding fragmentors, collision energies (CE) were optimized as follow: *m/z* 579.1→270.9 (fragmentor: 225 V, CE: 33 eV) for naringin, *m/z* 270.9→150.6 (fragmentor: 100 V, CE: 12 eV) for naringenin, and *m/z* 275.1→165.0 (fragmentor: 120 V, CE: 13 eV) for internal standard. The ion source parameters were Capillary 4000 V, Gas Flow 10 L/min, Nebulizer 25 psi, Gas Temperature 350°C for maximum sensitivity. Other MS parameters were adopted from the recommended values for the instrument.

### Metabolite Profiling

To profile the metabolites in urine and feces samples, an aliquot of 200 μL volume acetonitrile (containing stable isotope labeled internal standard [2′,3′,5′,6′-D_4_]-4,6,4′-trihydroxydihydroaurone) was added to 100 μL of urine or feces extract. Then, the sample was vortex-mixed for 3 min and centrifuged at 15,000 × *g* for 30 min at 25°C. Finally, 10 μL supernatant was used for UFLC-Q-TOF-MS/MS analysis.

Metabolite profiling was carried out with a Shimadzu UFLC XR system tandem a hybrid triple quadrupole time-of-flight mass spectrometer (Triple TOF^TM^ 5600 plus; AB Sciex, Foster City, CA, United States) equipped with an ESI source in negative-ionization mode. Chromatographic separation was performed using a Kinetex C_18_ column (3.0 × 150 mm, 2.6 μm, 100 Å; Phenomenex, Torrance, CA, United States) maintained at 40°C and eluted at a flow rate of 0.3 mL/min. The mobile phase 0.1% aqueous formic acid (*v/v*) (A) and 0.1% acidic methanol (*v/v*) (B) were applied to the following gradient elution program: B% linear gradient from 5% to 65% (0–10 min), 65–82.5% (10–20 min), maintained at 100% for 4 min (20.1–24.1 min), and then decreased bank and maintained for 5 min (25.0–30.0 min) at 5% to equilibrate.

The main instrumental conditions for Triple TOF^TM^ 5600 plus were set as follows: TOF-MS scan range was from *m/z* 100 to 1500, product ion scan range was from *m/z* 50 to 1500, ion source gas 1 and gas 2 were both 55 psi, curtain gas was 35 psi, ion source temperature was 550°C, ion spray voltage floating was -4,500 V, collision energy was 35 eV, collision energy spread was 25 eV, and declustering potential was 80 V. Nitrogen was used as nebulizer and auxiliary gas. Data acquisition was carried out using Analyst^®^ TF 1.6 software (AB Sciex, Foster City, CA, United States) in information-dependent acquisition (IDA) mode.

Metabolites identification was based on chromatographic elution time, chemical composition, MS/MS fragmentation pattern, and comparisons with available standards and references.

### Data Analysis

Calculation of pharmacokinetic parameters was performed using Drug and Statistics (DAS) software (Version 3.0, Shanghai University of Traditional Chinese Medicine; Shanghai, China) with a non-compartmental statistical model. Peak concentration (*C*_max_) and time to reach *C*_max_ (*T*_max_) were obtained from actual data. Data are expressed as mean ± standard error (SE) and were evaluated by Student’s *t*-tests in SPSS 18.0 (SPSS Inc., Chicago, IL, United States). *P*-values less than 0.05 or 0.01 were considered statistically significant. Figures were plotted with GraphPad Prism (Version 7.00) and Microsoft Excel (Version 2016).

## Results and Discussion

### Method Validation

Sharp and fine peaks were obtained for all analytes, and no interference was observed. Acceptable linearities were achieved in corresponding range for all analytes with correlation coefficients (*r*) > 0.99. Within-run precision for LLOQ were <20%, while that for low, middle, high QC were <15%. Between-run precision of these four concentration levels were all within 15%. RSD for matrix effects of each analyte at assigned concentration level are within 15%. These results (shown in Supplementary Table S1) indicated that developed methods were reliable and reproducible for the quantitative analysis.

### Pharmacokinetic Parameters

Rodents older than 20 months are commonly at a later stage of aging (Li and Gao, 2016; Gros and Wang, 2018). In this work, 20-month-old Sprague-Dawley rats were used as *in vivo* model to evaluate the ADME processes of naringin in aged animals. As reported in previous studies (Fang et al., 2006; Liu et al., 2012; Zou et al., 2012), after the oral administration of naringin, unaltered naringin was barely detected and naringenin glucuronide/sulfate conjugates were found to be predominant existing form in circulatory system. Thus, to quantify naringin and total naringenin, biological samples analyzed after hydrolysis with β-glucuronidase/sulfatase.

**Figure 1:**
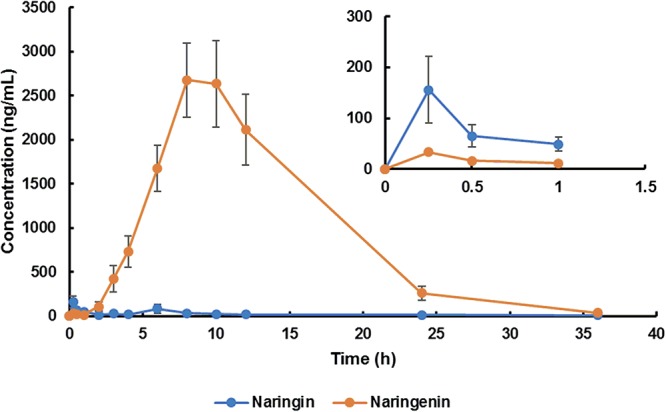
Plasma concentration-time curve of naringin and total naringenin in aged rats after a single oral administration of 42 mg/kg naringin.

The mean plasma concentration-time curve and pharmacokinetic parameters of naringin and total naringenin for aged rats were presented in Figure 1 and Table 1, respectively. Similar to reported pharmacokinetic studies concerning other flavonoid compounds (Li et al., 2012; Xiong et al., 2015), double-peak phenomenon was observed in the plasma profile of naringin and total naringenin after an oral administration of naringin, probably flowed from enterohepatic circulation or gastric emptying-regulated absorption (Deng et al., 2017). The concentration level of naringenin at the first peak was much lower than that at the second peak, revealed that just little naringin could be rapidly metabolized into naringenin and then absorbed into circulatory system. Most naringin was sequentially moved with gastrointestinal peristalsis, hydrolyzed to naringenin, and absorbed by gastrointestinal tract to construct the second peak, leading to a slow absorption of naringenin. Compared with adult rats (Wang et al., 2014; Ni et al., 2016), the *T*_max_ of naringenin was significantly delayed for about 3.5 h and the half washout time (*t*_1/2_) was prolonged for about 1.5 h. These differences probably resulted from age-related changes in the gastrointestinal tract (Corsonello et al., 2010) and hepatorenal function (Zheng et al., 2018), including the reduction of gastric emptying, gastrointestinal peristalsis, bowel surface area, and splanchnic blood flow. It is interesting to note that the *C*_max_ and area under the plasma concentration-time curve (AUC) were relatively higher (about two times) than that observed in adult rats (Wen et al., 2014). On the one hand, time extension of gastric emptying and gastrointestinal peristalsis may prolong the exposure time of naringin to gut and lead to a more complete absorption. On the other hand, metabolic and excretive elimination of naringin was likely to be weakened due to the decrease of splanchnic blood flow, cytochromes P450 activities (Zheng et al., 2018), and drug transporter expression (Joseph et al., 2015). These changes given rise to a higher exposure of naringin and naringenin in aged rats. In addition, there existed significant differences between the pharmacokinetic parameters obtained in female and male aged rats. As shown in Table 1, AUC (*P* < 0.05) and *t*_1/2_ (*P* < 0.01) of naringenin in female aged rats were significantly higher than that in male rats, while there shown no differences on *T*_max_ and *C*_max_. These results suggested that the elimination rate of naringenin in female aged rats was lower than that in male rats, probably associated with gender-related differences of cytochromes P450 (Scandlyn et al., 2008) and glomerular filtration rate (Xu et al., 2010).

**Table 1:** Pharmacokinetic parameters of naringin and total naringenin after a single oral administration of 42 mg/kg naringin in aged rats.

Parameters ^a^	Male + female		Male		Female	
	Naringin	Total naringenin	Naringin	Total naringenin	Naringin	Total naringenin ^b^
AUC_0-t_ (μg/L^∗^h)	459.6 ± 112.2	33115.2 ± 4849.9	528.9 ± 191.5	23021.6 ± 3860.2	390.3 ± 129.5	43208.7 ± 6916.6*
AUC_0-∞_ (μg/L^∗^h)	601.2 ± 133.0	33438.3 ± 4831.9	663.2 ± 211.0	23274.5 ± 3746.6	539.2 ± 178.2	43602.1 ± 6882.6*
*t*_1/2_ (h)	9.52 ± 2.76	3.18 ± 0.43	9.34 ± 3.56	2.02 ± 0.13	9.70 ± 4.58	4.33 ± 0.51**
*T*_max_ 1 (h)	0.50 ± 0.09	0.25 ± 0.00	0.54 ± 0.15	0.25 ± 0.00	0.46 ± 0.12	0.25 ± 0.00
*T*_max_ 2 (h)	5.20 ± 0.51	8.83 ± 0.52	4.75 ± 0.75	8.33 ± 0.61	5.50 ± 0.72	9.33 ± 0.84
*C*_max_ (μg/L)	179.6 ± 65.2	3520.6 ± 430.7	230.2 ± 129.8	2845.8 ± 491.0	128.9 ± 28.6	4195.3 ± 626.7

^a^*AUC, area under the plasma concentration-time curve; *t*_*1/2*_, half washout time; *T*_*max*_, time to reach *C*_*max*_; *C*_*max*_, peak concentration. ^*b*^Compared with male rats, ^∗^*P* < 0.05, ^∗∗^*P* < 0.01*.

### Tissue Distribution

**Figure 2:**
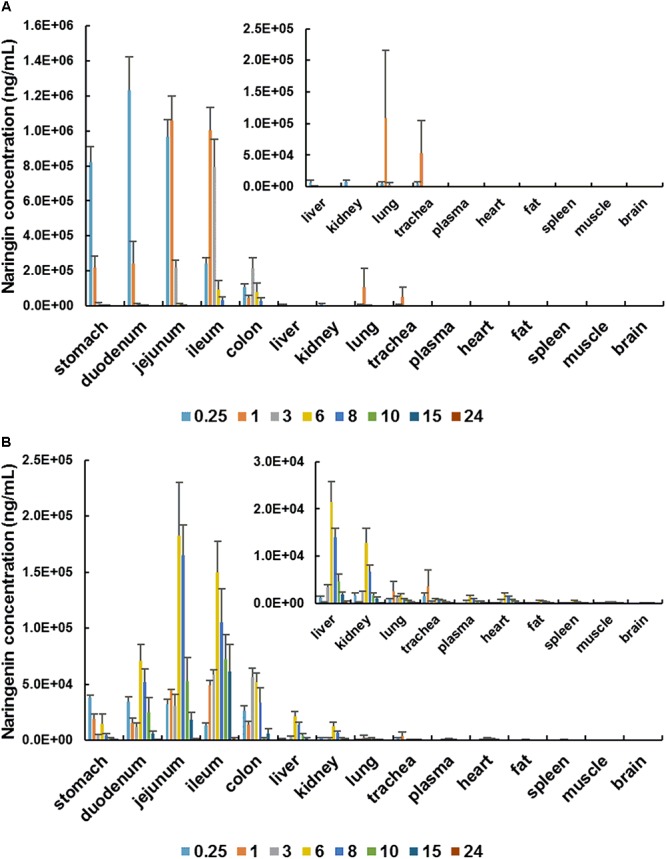
Tissue distribution profiles of naringin **(A)** and total naringenin **(B)** in aged rat at different time points after a single oral administration of 42 mg/kg naringin.

In the distribution study, the concentrations of naringin and total naringenin were determined in plasma and multiple tissues within 24 h after a single oral administration. Tissue distribution profiles of naringin and total naringenin in rat at different time points are charted in Figure 2. Obtained results indicated that naringin and its metabolites naringenin were widely distributed in gastrointestinal tract and major organs after single oral dose of naringin. The highest concentration of naringin and total naringenin in tested tissues mainly appeared within 3 h and 6 h, respectively. Compared with adult rats (Zou et al., 2012), there was a delay in the *T*_max_ of naringenin, revealing a slower absorption in aged rats. In addition to gastrointestinal tract, the accumulation of naringin in tissues was in descending order as follows (shown in Supplementary Table S2): lung > trachea > liver > kidney > muscle > fat > spleen > heart > brain, while that of total naringenin was: liver > kidney > lung > trachea > heart > fat > spleen > muscle > brain. These orders were significantly different with adult rats (Zou et al., 2012; Lin et al., 2014), showing age-associated changes in tissue distribution of naringin.

Naringin has been proved to be an effective peripheral antitussive and expectorant. It significantly inhibits the responses of rapidly adapting receptors (RARs), which can evoke cough reflex and distribute in lung and trachea (Gao et al., 2011). Naringin reduce sputum by inhibiting the transformation and proliferation of airway goblet cells, as well as the generation of airway mucin (Lin et al., 2008). Thus, lung and trachea are considered as the main target organ of naringin in relieving respiratory disease (Liu et al., 2012; Li et al., 2015). Compared with adult rats (Zou et al., 2012), the accumulation of naringin and naringenin in lung and trachea were higher in aged rats. Therefore, naringin may more effectively to relieve cough and sputum in aged individuals. In contrast, spleen, fat, muscle, and brain were found to be poorly distributed tissues for naringin and its metabolites. The concentration levels of naringin and total naringenin in brain were much lower than that in plasma, suggesting the limited impacts of naringin on central nervous system. What is more, naringin and naringenin were found to be few within 24 h in all examined tissues, showing that there exist no long-term accumulations in tissues.

**Figure 3:**
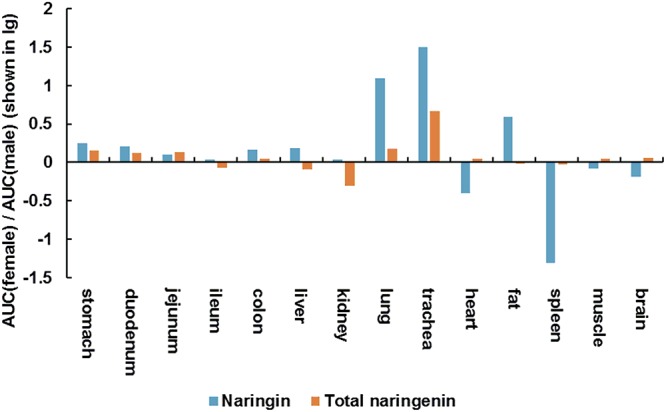
AUC (female)/AUC (male) (shown in lg) of naringin and total naringenin in tissues collected from aged rats.

As shown in Figure 3, there shown minor differences in AUC of naringin and total naringenin obtained in stomach, gastrointestinal tract, liver, kidney, muscle, brain between female and male aged rats. It should be noted that AUC of naringin in lung (1,250%) and trachea (3,140%) collected from female rats were significantly higher than that in male rats, suggesting that naringin may show gender differences in the treatment of respiratory diseases in elderly individuals.

### Metabolite Profiling of Naringin

**Figure 4:**
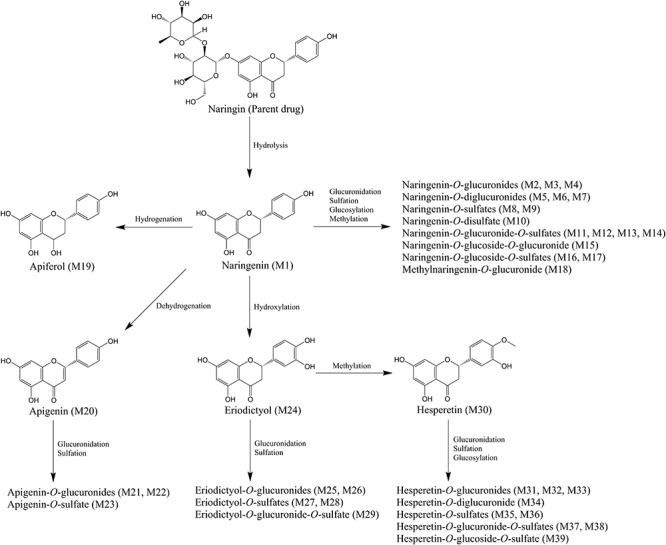
Proposed metabolic pathways of flavonoid metabolites in aged rats after a single oral dose of 42 mg/kg naringin. The number of metabolites in the bracket was corresponding to that in Supplementary Table S3.

Generally, the hydrolysis of flavonoids glycosides was identified as the first and determinant step in the absorption (Chen et al., 2014; Zeng et al., 2017b). In this study, free naringin was barely detected, aligned with previous results (Wang et al., 2006; Lin et al., 2014). After the hydrolysis, naringenin subsequently involved in hydrogenation, dehydrogenation, hydroxylation, and methylation to yield several other aglycons, including apiferol, apigenin, eriodictyol, and hesperetin. Moreover, naringenin and generated aglycons extensively engaged in glucuronidation and sulfation catalyzed by phase II metabolic enzymes in gastrointestinal tract, liver, and other tissues (Wang et al., 2006; Liu et al., 2012; Joshi et al., 2018). As shown in Supplementary Table S3, a total of 39 flavonoid metabolites—comprising *O*-glucuronide, *O*-diglucuronide, *O*-sulfate, *O*-disulfate, *O*-glucuronide-sulfate, *O*-glucoside-*O*-glucuronide, *O*-glucoside-*O*-sulfate derivatives of naringenin, apigenin, eriodictyol, and hesperetin were identified or partially identified in urine and/or feces samples. The proposed metabolic pathways of above metabolites were illustrated in Figure 4. For these flavonoid metabolites, typical Retro Diels-Alder (RDA) reactions were observed in the MS/MS fragmentation (shown in Table 2), in line with reported results (Fabre et al., 2001; Ma et al., 2015; Zeng et al., 2017a). For example, the fragment ions of naringenin at *m/z* 151 and 119 were yielded by RDA reaction, which could be taken as diagnostic characters for naringenin.

**Table 2:** Accumulative excretion (% dose) of naringin, naringenin, and total in urine and feces after a single oral administration of 42 mg/kg naringin to aged rats.

Accumulative excretion (% dose)	Male + female	Male	Female ^a^
Urine			
Naringin	4.9E-03 ± 2.1E-03	2.5E-03 ± 5.9E-04	7.2E-03 ± 4.0E-03
Naringenin	19.0 ± 2.9	12.7 ± 2.6	25.3 ± 3.6*
Urinary total	93.4 ± 9.4	84.9 ± 16.9	101.9 ± 8.7
Feces			
Naringin	1.7E-03 ± 7.8E-04	6.4E-04 ± 4.2E-04	2.7E-03 ± 1.2E-03
Naringenin	3.7E-04 ± 7.5E-05	2.7E-04 ± 1.1E-04	4.5E-04 ± 9.6E-05
Fecal total	4.7 ± 3.9	0.7 ± 0.3	8.8 ± 7.7

^a^*Compared with male aged rats. ^∗^*P* < 0.05*.

**Figure 5:**
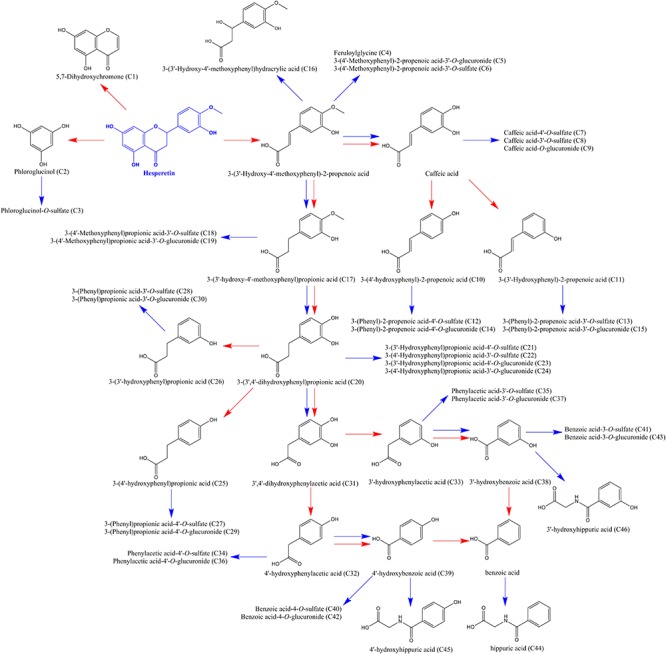
Proposed pathways for the catabolism of hesperetin by intestinal microbiota and phase II enzymes in aged rats after a single oral dose of 42 mg/kg naringin. Red arrows represent microbiota-mediated steps, while blue arrows indicate rat enzyme-mediated conversions. The number of metabolites in the bracket was corresponding to that in Supplementary Table S4.

Meanwhile, unabsorbed flavonoids, as well as the metabolites excreted by enterohepatic circulation (Zhang et al., 2007), further undergo microbiota-mediated ring fission and yield a family of phenolic catabolites (Zou et al., 2015; Zeng et al., 2018b). In this study, a total of 46 microbial-derived phenolic catabolites, such as phenylpropenoic acid, phenylpropionic acid, phenylacetic acid, benzoic acid, benzenetriol and benzoylglycine derivatives, including free phenolics and phase II sulfate, glucuronide metabolites were detected (detailed information were presented in Supplementary Table S4). As Pereira-Caro et al. (2016) described, intestinal microbiota plays a vital role in the rupture of flavonoid skeleton, while subsequent glucuronide, sulfate, and glycine conjugation are mammalian in origin. Demethylation and hydrogenation can be catalyzed by both microbial and mammalian enzymes, yet dehydroxylation is almost certainly mediated by the gut microbiota. For example, with the ring fission, hesperetin was catabolized into phloroglucinol and 3-(3′-hydroxy-4′-methoxyphenyl)-2-propenoic acid. The latter catabolite was converted to caffeic acid, 3-(3′-hydroxy)-2-propenoic acid, and 3-(4′-hydroxy)-2-propenoic acid by demethylation and subsequent dehydroxylation. Meanwhile, 3-(3′-hydroxy-4′-methoxyphenyl)-2-propenoic acid was hydrogenated, demethylated, and dehydroxylated, yielding 3-(3′-hydroxy-4′-methoxyphenyl)propionic acid, 3-(3′,4′-dihydroxyphenyl) propionic acid, 3-(3′-hydroxyphenyl)propionic acid, and 3-(4′-hydroxyphenyl) propionic acid. It is worth to note that 3-(4′-hydroxyphenyl)propionic acid was considered as a potential biomarker of the intake of naringin in prior studies (Liu et al., 2012; Zou et al., 2015). 3-(3′,4′-dihydroxyphenyl)propionic acid was finally degraded to benzoic acid via successive dehydroxylation and shortening of the side chain. Generated phenolic acids were further converted to glucuronide, sulfate, and glycine derivates by phase II enzymes. In addition, 3-(4′-hydroxyphenyl)propionic acid could be catabolized to 4′-hydroxybenzoic acid and benzoic acid, contributed to the accumulation of 4’-hydroxyhippuric acid and hippuric acid, similar to 3-(3′-hydroxyphenyl)propionic acid. The proposed pathways for the catabolism of hesperetin by intestinal microbiota and phase II enzymes were illustrated in Figure 5.

Benefited from the high sensitivity and resolution of UFLC-Q-TOF-MS/MS system, more flavonoid metabolites (including *O*-diglucuronides, *O*-disulfate, *O*-glucuronide-*O*-sulfates, *O*-glucoside*-O*-glucuronides, *O*-glucoside*-O*-sulfates of naringenin and hesperetin) and phenolic catabolites (mainly derivates of 3-(phenyl)-2-propenoic acid) were screened in the urine and feces samples collected from aged rats, comparing with reported data in adult rats (Wang et al., 2006; Liu et al., 2012). Although remarkable age-related decrease in uridine 5’-diphospho-glucuronosyltransferase (UDP-glucuronosyltransferases) was observed in rats (Zheng et al., 2018), glucuronidation still plays an important role in the metabolism of naringin in aged rats, as well as sulfation.

### Urinary and Fecal Excretion

The volumes of urine collected from 12 aged rats during each time period were presented in Supplementary Table S5, as well as the weight of feces samples (shown in Supplementary Table S6).

**Figure 6:**
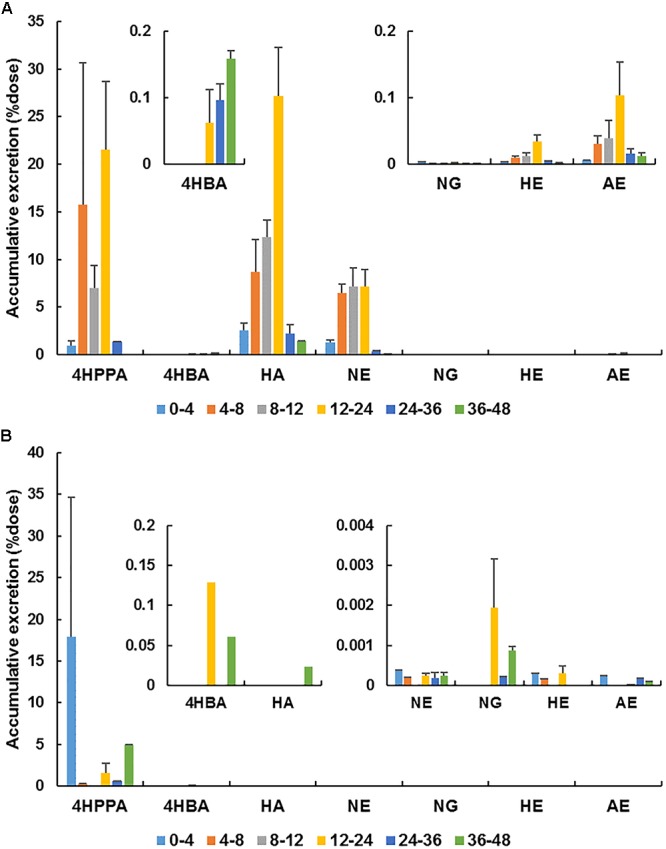
Accumulative excretion of naringin (NG) and its main metabolites [naringenin (NE), hesperetin (HE), apigenin (AE), hippuric acid (HA), 4-hydroxybenzonic acid (4HBA), and 3-(4′-hydroxyphenyl)propionic acid (4HPPA)] in urine **(A)** and feces **(B)** after a single oral administration of 42 mg/kg naringin to aged rats.

Based on the results of metabolite profiling, the concentrations of naringin and its main metabolites [naringenin, apigenin, hesperetin, hippuric acid, 4-hydroxybenzonic acid, and 3-(4’-hydroxyphenyl)propionic acid] were determined in rat urine and feces collected within 48 h after the oral administration of naringin. The MRM parameters for these metabolites were set as follows: *m/z* 301.0→163.9 (fragmentor: 195 V, CE: 19 eV) for hesperetin, *m/z* 269.0→150.6 (fragmentor: 150 V, CE: 20 eV) for apigenin, *m/z* 137.0→93.0 (fragmentor: 80 V, CE: 12 eV) for 4-hydroxybenzoic acid, *m/z* 165.0→121.1 (fragmentor: 90 V, CE: 7 eV) for 3-(4’-hydroxyphenyl) propionic acid, and *m/z* 178.0→134.0 (fragmentor: 100 V, CE: 7 eV) for hippuric acid. The cumulative excretion profiles of naringin and its main metabolites are presented in Figure 6. Unmetabolized naringin were barely detected in urine (0.01% dose) and feces (0.03‰ dose), revealed that naringin undergo extensive metabolism in aged rats. Naringenin, hippuric acid, and 3-(4’-hydroxyphenyl)propionic acid were found to be the predominated metabolites in urine, while only 3-(4’-hydroxyphenyl)propionic acid was abundant in feces. As shown in Figure 6, most metabolites were excreted within 36 h post dose.

Aging is generally associated with reduced kidney function (Ren et al., 2015). In the present work, the urinary excretion rate of naringenin was high within 4–12 h post dose and total naringenin was excreted within 24 h in aged rats, showing a delay compared with adult rats (4–8 h) (Liu et al., 2012). These differences properly associated with the reduction of kidney function in aged rats. However, taking naringin and naringenin together, the total cumulative excretions in urine amounted to 19.0% dose in aged rats, significantly higher than that in adult rats (0.9%) (Liu et al., 2012). Given the increase of plasma exposure for naringin and naringenin, the decrease of kidney function was unfortunately covered. Additionally, the urinary accumulative excretion of naringenin in female aged rats (25.3%) was remarkedly higher than that in male rats (12.7%) (*P* < 0.05), probably flowed from the higher plasma exposure of naringenin in female aged rats. The total cumulative excretions of naringin and total naringenin in feces collected from aged rats (0.02‰ dose) dose were substantially lower than that in adult rats (4.7% dose) (Liu et al., 2012), reinforcing that naringin underwent more complete metabolism and absorption in aged rats.

As presented in Table 2, taking naringin and its main metabolites into account, the total accumulative excretion in urine was 93.4%, while that in feces was 4.7%. However, when counting the molar concentrations of above seven compounds together, the urinary cumulative excretion was found to exceed the dose in four aged rats although the blank value was deducted (shown in Supplementary Figure S1). Urinary excretion of naringenin, hippuric acid, and 3-(4’-hydroxyphenyl)propionic acid were all increased after intake of naringin. Naringenin doubtlessly derived from ingested naringin, but hippuric acid and 3-(4’-hydroxyphenyl)propionic acid could be generated in intrinsic metabolic processes. Specifically, hippuric acid is probably derived from endogenous catecholamine (Pero, 2010), while 3-(4’-hydroxyphenyl)propionic acid is a major metabolite of tyrosine (Feng et al., 2014). Therefore, without other methodologies, it is difficult to determine the exact amounts of above phenolic catabolites derived from ingested naringin. Recently, we are developing a stable isotope labeling method to address this issue.

## Conclusion

In the present study, a series of sensitive, rapid, and reliable RRLC-QQQ-MS/MS methods were established, validated, and applied to the quantification of naringin and naringenin in plasma, urine, feces, and various tissue samples collected from aged rats. After a single oral administration of naringin, the pharmacokinetic parameters were calculated and a higher exposure of naringin and naringenin were observed in aged rats. Naringin and naringenin were mostly distributed in gastrointestinal tract, liver, kidney, lung, and trachea. With UFLC-Q-TOF-MS/MS, a total of 39 flavonoid metabolites (mainly glucuronides and sulfates) and 46 microbial-derived phenolic catabolites were identified or partially identified in urine and/or feces samples collected post dose. The metabolism pathways of naringin in aged rats were proposed. Naringenin, hippuric acid, and 3-(4’-hydroxyphenyl)propionic acid were found to be the predominated metabolites using a RRLC-QQQ-MS/MS method. This study systemically investigated the pharmacokinetics, tissue distribution, metabolism, and excretion properties of naringin in aged rats and revealed age- and gender-related changes in the *in vivo* behavior of naringin. These results would be helpful for the interpretation of pharmacokinetics and pharmacodynamics of naringin in aged population.

## Author Contributions

XZ, WS, and HY conceived and designed the study. XZ, WS, WP, and HY analyzed the research data and wrote the manuscript. XZ, YZ, YdH, YaH, and HR carried out the experiments.

## Conflict of Interest Statement

The authors declare that the research was conducted in the absence of any commercial or financial relationships that could be construed as a potential conflict of interest.
