# Supplementary material for: Pharmacokinetics, Tissue Distribution, Metabolism, and Excretion of Naringin in Aged Rats
Source: Front Pharmacol. 2019 Jan 28;10:34. doi: 10.3389/fphar.2019.00034 (PMC6362423; doi:10.3389/fphar.2019.00034)
Supplement: Supplementary file 1 [file Data_Sheet_1.pdf]

Supplementary Materials

Journal name

Frontiers in Pharmacology

Article title

Pharmacokinetics, tissue distribution, metabolism, and excretion of naringin in aged rats

Contents

1. Table S1 *(Page 2-3)*

2. Table S2 *(Page 4)*

3. Table S3 *(Page 5-9)*

4. Table S4 *(Page 10-14)*

5. Table S5 *(Page 15)*

6. Table S6 *(Page 15)*

7. Figure S1 *(Page 16)*

Table S1. Results of method validation for the determination of naringin (NG), naringenin (NE), hesperetin (HE), apigenin (AE), hippuric acid (HA), 4-hydroxybenzonic acid (4HBA), and 3-(4'-hydroxyphenyl)propionic acid (4HPPA) in biological samples.

**Matrix**	**Compounds**	**Calibration curve range (ng/mL)**	**Linearity equations**	**r**	**Within-run precisions (%)(n=6)**		**Between-run precisions (%)(n=18)**	**Coefficient of variation of matrix factors (%) (n=6)**	
					**LLOQ [a]**	**L, M, H [b]**	**LLOQ, L, M, H**	**L**	**H**
Plasma	NG	4.820~1928	Y=0.0743*X+2.057E-005	0.9979	10.6~17.2	5.2~8.6	6.2~14.8	2.9	0.8
	NE	5.450~2180	Y=0.0881*X-8.353E-005	0.9986	5.8~13.1	3.0~14.2	4.6~12.9	4.3	2.2
Stomach	NG	9.640~3856	Y=0.1404*X-1.248E-004	0.9950	4.2~8.5	3.0~9.5	3.9~7.0	11.9	9.5
	NE	5.450~2180	Y=0.3253*X-1.504E-004	0.9944	3.4~7.9	2.1~6.2	2.9~6.3	10.4	12.7
Intestines	NG	9.640~3856	Y=0.2757*X-7.658E-004	0.9939	4.3~10.6	0.6~3.1	2.3~7.9	2.1	13.1
	NE	5.450~2180	Y=0.2724*X-8.390E-005	0.9982	5.3~11.3	2.1~5.9	3.5~8.2	13.8	9.9
Liver	NG	4.820~964.0	Y=0.1958*X+9.865E-004	0.9977	9.4~12.4	1.3~10.2	5.9~11.0	5.4	8.7
	NE	5.450~1090	Y=0.1849*X-2.139E-004	0.9959	8.8~11.9;	2.9~12.9	8.6~11.4	3.7	8.5
Kidney	NG	2.410~482.0	Y=0.5159*X-0.0011	0.9968	11.6~14.9	2.1~12.0	2.4~13.9	5.4	3.3
	NE	5.450~1090	Y=0.7359*X-0.0048	0.9926	7.2~15.1	2.3~9.4	3.7~10.9	9.4	7.9
Trachea	NG	4.820~482.0	Y=0.0484*X-4.888E-005	0.9951	11.0~14.8	2.8~13.0	8.7~14.7	8.7	4.2
	NE	2.725~272.5	Y=0.3431*X-0.0012	0.9980	1.9~9.4	7.8~12.3	7.3~11.1	9.0	12.8
Lung	NG	2.410~241.0	Y=0.1125*X-8.2494E-004	0.9961	7.0~11.1	1.9~10.0	3.0~11.0	4.4	2.6
	NE	2.725~272.5	Y=0.4211*X-0.0012	0.9986	5.1~13.5	2.3~8.1	4.1~10.0	13.6	13.8
Heart	NG	2.410~241.0	Y=0.1399*X+5.7987E-004	0.9947	11.1~13.9	2.3~13.7	6.7~11.9	5.4	1.4
	NE	2.725~272.5	Y=0.1780*X+0.0010	0.9965	6.2~11.1	5.5~10.0	7.2~10.6	9.2	10.6
Spleen	NG	2.410~241.0	Y=0.1361*X-6.2228E-005	0.9989	9.6~18.3	2.0~10.6	3.0~14.5	4.0	2.0
	NE	2.725~272.5	Y=0.4076*X-9.9717E-004	0.9953	6.1~9.2	3.2~9.7	5.4~8.2	13.2	14.8
Muscle	NG	2.410~241.0	Y=0.1143*X+4.4749E-004	0.9954	6.8~19.4	6.1~12.0	8.1~14.8	1.5	1.5
	NE	2.725~272.5	Y=0.4584*X-0.0016	0.9962	7.8~10.8	1.9~9.2	5.1~8.9	4.6	2.5

Fat	NG	2.410~241.0	Y=0.0188*X+1.0842E-004	0.9987	10.5~14.5	2.9~8.8	5.3~12.7	4.4	7.7
	NE	2.725~272.5	Y=0.0132*X-2.8570E-005	0.9960	4.3~18.9	2.7~10.3	3.4~12.2	14.3	13.0
Brain	NG	2.410~241.0	Y=0.1334*X+5.1398E-004	0.9950	6.6~17.2	3.5~11.2	7.6~12.6	2.4	0.3
	NE	2.725~272.5	Y=0.3706*X+2.1430E-005	0.9976	5.9~18.3	1.1~11.9	3.2~14.1	11.6	4.6
Urine	NG	2.470~988.0	Y=129.5*X+303.6	0.9993	10.1~12.7	1.8~11.2	7.1~11.0	9.0	5.8
	NE	26.80~10720	Y=115.5*X-758.0	0.9989	4.6~6.0	0.9~6.4	3.6~5.5	9.4	7.4
	HE	2.427~970.9	Y=24.70*X+4.700	0.9986	6.8~12.2	2.5~14.6	5.9~12.3	5.6	5.4
	AE	12.46~4986	Y=39.20*X+79.00	0.9995	6.4~9.1	1.3~7.5	2.3~8.2	3.4	5.8
	HA	5575~55750	Y=2621.5*X+54215.3	0.9966	5.9~10.3	2.2~10.0	4.9~9.7	13.8	8.2
	4HBA	1087~10870	Y=2368.3*X+2391.1	0.9979	3.6~11.5	2.7~8.9	5.5~8.9	6.3	9.7
	4HPPA	9996~99960	Y=911.5*X+25749.8	0.9960	5.2~10.9	2.4~9.9	5.2~8.2	5.9	8.6
Feces	NG	1.976~197.6	Y=9.732E-006*X+1.031E-004	0.9989	8.2~12.0	4.7~11.0	8.0~11.4	8.9	5.8
	NE	2.144~214.4	Y=1.406E-004*X-9.754E-005	0.9960	4.1~14.3	1.7~9.3	3.2~9.0	4.6	3.0
	HE	1.942~194.2	Y=1.441E-005*X-9.553E-006	0.9989	9.0~9.7	2.5~11.1	3.9~10.0	13.3	3.3
	AE	0.9972~99.72	Y=4.950E-005*X+1.520E-005	0.9980	8.8~10.0	2.5~10.9	5.4~10.2	8.3	2.6
	HA	278.8~2788	Y=0.0085*X+3.044E-004	0.9985	4.3~10.2	1.7~10.4	5.7~7.9	1.5	2.2
	4HBA	271.8~2718	Y=0.0075*X+2.078E-004	0.9954	4.8~10.1	3.6~10.2	5.6~8.6	5.3	3.5
	4HPPA	2499~24990	Y=0.0032*X+0.0167	0.9932	9.0~10.2	3.4~8.7	6.6~9.9	2.9	5.0

[a] LLOQ, lower limit of quantification.

[b] L, M, H represent low, middle, high QC concentration levels, respectively.

Table S2. AUC, C_{max} and T_{max} for naringin and total naringenin in tissues after a single oral administration of 42 mg/kg naringin to aged rats.

**Tissues**	**Naringin**			**Total naringenin**		
	**AUC (ng*h/g)**	**C_{max} (ng/g)**	**T_{max} (h)**	**AUC (ng*h/g)**	**C_{max} (ng/g)**	**T_{max} (h)**
Stomach	658476	820478	0.25	111641	38431	0.25
Duodenum	840117	1232198	0.25	473033	70936	6
Jejunum	2404138	1059446	1	1251818	182364	6
Ileum	3761105	1003811	1	1490177	149457	6
Colon	895386	214840	3	411298	56032	3
Liver	5879	7087	0.25	121958	21423	6
Kidney	5286	7691	0.25	65837	12706	6
Lung	161811	108925	1	16294	2538	1
Trachea	80225	52876	1	13826	3623	1
Heart	1070	211.9	0.25	13530	1426	6
Fat	1589	159.9	10	4308	490.3	6
Spleen	1152	181.0	0.25	3163	479.7	6
Muscle	1799	358.9	0.25	2306	291.3	6
Brain	1040	222.9	10	913.0	75.9	6

Table S3. UFLC-Q-TOF-MS/MS based identifications of flavonoid metabolites urine and feces samples collected 0-48 h after a single oral administration of naringin in aged rats.

No.	Identified metabolites	Formula	RT (min)	$[M-H]^-$ (Error, ppm)	Fragment ions in negative mode [a]	Source
Parent drug	Naringin [b]	$C_{27}H_{32}O_{14}$	11.4	579.1717 (-0.4)	459.1196$[M-H-C_8H_8O]^-$, 313.0713$[M-H-C_8H_8O-Rha]^-$, 271.0601$[M-H-Rha-Glc]^-$, 151.0024$[M-H-Rha-Glc-C_8H_8O]^-$	Urine, Feces
M1	Naringenin [b]	$C_{15}H_{12}O_5$	13.7	271.0617 (1.8)	177.0202$[M-H-C_6H_6O]^-$, 151.0043$[M-H-C_8H_8O]^-$, 119.0512$[M-H-C_7H_4O_4]^-$, 107.0150$[M-H-C_8H_8O-CO_2]^-$, 93.0366$[M-H-C_9H_6O_4]^-$, 83.0147	Urine, Feces
M2	Naringenin-5-*O*-glucuronide	$C_{21}H_{20}O_{11}$	10.7	447.0932 (-0.2)	271.0610$[M-H-GlcUA]^-$, 175.0251$[M-H-NE]^-$, 151.0037$[M-H-GlcUA-C_8H_8O]^-$, 113.0259$[M-H-NE-CO_2-H_2O]^-$	Urine
M3	Naringenin-7-*O*-glucuronide [b]	$C_{21}H_{20}O_{11}$	11.5	447.0941 (1.8)	271.0611$[M-H-GlcUA]^-$, 227.0715, 177.0191$[M-H-GlcUA-C_6H_6O]^-$, 175.0239$[M-H-NE]^-$, 151.0034$[M-H-GlcUA-C_8H_8O]^-$, 119.0509$[M-H-GlcUA-C_7H_4O_4]^-$, 113.0251$[M-H-NE-CO_2-H_2O]^-$, 85.0318$[M-H-NE-CO_2-H_2O-CO]^-$	Urine
M4	Naringenin-4'-*O*-glucuronide [b]	$C_{21}H_{20}O_{11}$	11.7	447.0942 (2.1)	271.0614$[M-H-GlcUA]^-$, 177.0197$[M-H-GlcUA-C_6H_6O]^-$, 175.0252$[M-H-NE]^-$, 151.0046$[M-H-GlcUA-C_8H_8O]^-$, 119.0516$[M-H-GlcUA-C_7H_4O_4]^-$, 113.0258$[M-H-NE-CO_2-H_2O]^-$, 85.0320$[M-H-NE-CO_2-H_2O-CO]^-$	Urine
M5	Naringenin-4′,7-*O*-diglucuronide	$C_{27}H_{28}O_{17}$	8.7	623.1247 (-1.2)	447.0915$[M-H-GlcUA]^-$, 313.0691$[M-H-GlcUA-C_4H_6O_5]^-$, 271.0600$[M-H-2GlcUA]^-$, 175.0236$[M-H-NE-GlcUA]^-$, 151.0033$[M-H-2GlcUA-C_8H_8O]^-$, 113.0240$[M-H-NE-GlcUA-CO_2-H_2O]^-$, 107.0143$[M-H-2GlcUA-C_8H_8O-CO_2]^-$, 85.0310$[M-H-NE-GlcUA-CO_2-H_2O-CO]^-$	Urine
M6	Naringenin-5,7-*O*-diglucuronide	$C_{27}H_{28}O_{17}$	9.4	623.1257 (0.6)	447.0943$[M-H-GlcUA]^-$, 271.0610$[M-H-2GlcUA]^-$, 175.0223$[M-H-NE-GlcUA]^-$, 151.0027$[M-H-2GlcUA-C_8H_8O]^-$, 113.0224$[M-H-NE-GlcUA-CO_2-H_2O]^-$	Urine
M7	Naringenin-4′,5-*O*-diglucuronide	$C_{27}H_{28}O_{17}$	10.8	623.1240 (-2.2)	447.0939$[M-H-GlcUA]^-$, 313.0705$[M-H-GlcUA-C_4H_6O_5]^-$, 271.0618$[M-H-2GlcUA]^-$, 175.0244$[M-H-NE-GlcUA]^-$, 151.0033$[M-H-2GlcUA-C_8H_8O]^-$,	Urine

					113.0254[M-H-NE-GlcUA-CO_2-H_2O]$^-$	
M8	Naringenin-4’-*O*-sulfate	$C_{15}H_{12}O_8S$	10.0	351.0181 (0.2)	271.0610[M-H-SO_3]$^-$, 177.0182[M-H-SO_3-C_6H_6O]$^-$, 165.0180[M-H-SO_3-C_7H_6O]$^-$, 151.0038[M-H-SO_3-C_8H_8O]$^-$, 119.0510[M-H-SO_3-$C_7H_4O_4$]$^-$, 107.0158[M-H-SO_3-C_8H_8O-CO_2]$^-$, 93.0366[M-H-SO_3-$C_9H_6O_4$]$^-$, 83.0152	Urine
M9	Naringenin-7-*O*-sulfate	$C_{15}H_{12}O_8S$	12.1	351.0186 (1.7)	271.0608[M-H-SO_3]$^-$, 177.0185[M-H-SO_3-C_6H_6O]$^-$, 151.0035[M-H-SO_3-C_8H_8O]$^-$, 119.0508[M-H-SO_3-$C_7H_4O_4$]$^-$, 107.0150[M-H-SO_3-C_8H_8O-CO_2]$^-$, 93.0356[M-H-SO_3-$C_9H_6O_4$]$^-$, 83.0149	Urine
M10	Naringenin-*O*-disulfate	$C_{15}H_{12}O_{11}S_2$	12.2	430.9744 (-1.0)	351.0169[M-H-SO_3]$^-$, 271.0599[M-H-2SO_3]$^-$, 177.0185[M-H-2SO_3-C_6H_6O]$^-$, 151.0048[M-H-2SO_3-C_8H_8O]$^-$, 119.0507[M-H-2SO_3-$C_7H_4O_4$]$^-$, 96.9621, 93.0341[M-H-2SO_3-$C_9H_6O_4$]$^-$	Urine
M11	Naringenin-*O*-glucuronide-*O*-sulfate	$C_{21}H_{20}O_{14}S$	7.6	527.0483 (-3.3)	447.0929[M-H-SO_3]$^-$, 351.0202[M-H-GlcUA]$^-$, 286.0708, 271.0599[M-H-SO3-GlcUA]$^-$, 175.0195[M-H-SO_3-NE]$^-$, 151.0062[M-H-SO_3-GlcUA-C_8H_8O]$^-$, 113.0226[M-H-SO_3-NE -CO_2-H_2O]$^-$, 85.0328[M-H-SO_3-NE -CO_2-H_2O-CO]$^-$	Urine
M12	Naringenin-*O*-glucuronide-*O*-sulfate	$C_{21}H_{20}O_{14}S$	8.0	527.0491 (-1.9)	447.0923[M-H-SO_3]$^-$, 351.0170[M-H-GlcUA]$^-$, 271.0604[M-H-SO3-GlcUA]$^-$, 177.0188[M-H-SO_3-GlcUA-C_6H_6O]$^-$, 175.0236[M-H-SO_3-NE]$^-$, 151.0022[M-H-SO_3-GlcUA-C_8H_8O]$^-$, 113.0245[M-H-SO_3-NE -CO_2-H_2O]$^-$, 96.9608, 85.0310[M-H-SO_3-NE -CO_2-H_2O-CO]$^-$	Urine
M13	Naringenin-*O*-glucuronide-*O*-sulfate	$C_{21}H_{20}O_{14}S$	8.9	527.0484 (-3.3)	447.0945[M-H-SO_3]$^-$, 351.0177[M-H-GlcUA]$^-$, 313.0721[M-H-SO_3-$C_4H_6O_5$]$^-$, 271.0605[M-H-SO_3-GlcUA]$^-$, 254.0707, 227.0710, 175.0243[M-H-SO_3-NE]$^-$, 165.0182[M-H-SO_3-GlcUA-C_7H_6O]$^-$, 151.0022[M-H-SO_3-GlcUA-C_8H_8O]$^-$, 119.0502[M-H-SO_3-GlcUA-$C_7H_4O_4$]$^-$, 113.0243[M-H-SO_3-NE -CO_2-H_2O]$^-$, 96.9606, 85.0334[M-H-SO_3-NE -CO_2-H_2O-CO]$^-$	Urine
M14	Naringenin-*O*-glucuronide-*O*-sulfate	$C_{21}H_{20}O_{14}S$	10.1	527.0486 (-2.9)	447.0936[M-H-SO_3]$^-$, 351.0183[M-H-GlcUA]$^-$, 271.0611[M-H-SO_3-GlcUA]$^-$, 175.0253[M-H-SO_3-NE]$^-$, 151.0043[M-H-SO_3-GlcUA-C_8H_8O]$^-$, 113.0258[M-H-SO_3-NE -CO_2-H_2O]$^-$, 85.0324[M-H-SO_3-NE -CO_2-H_2O-	Urine

					$CO]^-$	
M15	Naringenin-*O*-glucoside-*O*-glucuronide	$C_{27}H_{30}O_{16}$	8.8	609.1456 (-0.8)	447.0935[M-H-Glc]$^-$, 433.1141[M-H-GlcUA]$^-$, 402.1669[M-H-Glc-COOH]$^-$, 313.0712[M-H-Glc-$C_4H_6O_5$]$^-$, 271.0594[M-H-Glc-GlcUA]$^-$, 151.0000[M-H-Glc-GlcUA-C_8H_8O]$^-$, 113.0237[M-H- Glc-NE-CO_2-H_2O]$^-$	Urine
M16	Naringenin-*O*-glucoside-*O*-sulfate	$C_{21}H_{22}O_{13}S$	9.0	513.0710 (0.4)	433.1135[M-H-SO_3]$^-$, 358.9718, 313.0564[M-H-SO_3-$C_4H_8O_4$]$^-$, 271.0600[M-H-SO_3-Glc]$^-$, 151.0018[M-H-SO_3-Glc-C_8H_8O]$^-$, 119.0491[M-H-SO_3-Glc-$C_7H_4O_4$]$^-$	Urine
M17	Naringenin-*O*-glucoside-*O*-sulfate	$C_{21}H_{22}O_{13}S$	9.4	513.0703 (-1.1)	433.1145[M-H-SO_3]$^-$, 313.0560[M-H-SO_3-$C_4H_8O_4$]$^-$, 271.0603[M-H-SO_3-Glc]$^-$, 177.0197[M-H-SO_3-Glc-C_6H_6O]$^-$, 151.0030[M-H-SO_3-Glc-C_8H_8O]$^-$, 119.0500[M-H-SO_3-Glc-$C_7H_4O_4$]$^-$, 107.0137[M-H-SO_3-Glc-C_8H_8O-CO_2]$^-$	Urine
M18	Methylnaringenin-*O*-glucuronide	$C_{22}H_{22}O_{11}$	13.5	461.1087 (-0.4)	443.1747[M-H-H_2O]$^-$, 285.0765[M-H-GlcUA]$^-$, 175.0232[M-H-MNE]$^-$, 164.0098, 151.0022[M-H-GlcUA-$C_9H_{10}O$]$^-$, 113.0248[M-H-MNE-CO_2-H_2O]$^-$, 96.9612, 85.0326[M-H-MNE-CO_2-H_2O-CO]$^-$	Urine
M19	Apiferol	$C_{15}H_{14}O_5$	13.8	273.0765 (-1.2)	167.0352, 153.0089[M-H-C_8H_8O]$^-$, 123.0448, 119.0504[M-H-$C_7H_6O_4$]$^-$, 93.0345[M-H-$C_9H_8O_4$]$^-$	Urine
M20	Apigenin [b]	$C_{15}H_{10}O_5$	15.0	269.0455 (-0.3)	251.1606[M-H-H_2O]$^-$, 225.1405, 151.0065[M-H-C_8H_6O]$^-$, 117.0306[M-H-$C_7H_4O_4$]$^-$, 98.9536[M-H-$C_7H_4O_4$-H_2O]$^-$	Urine, Feces
M21	Apigenin-*O*-glucuronide	$C_{21}H_{18}O_{11}$	11.1	445.0772 (-1.0)	269.0440[M-H-GlcUA]$^-$, 175.0234[M-H-AE]$^-$, 113.0242[M-H-AE-CO_2-H_2O]$^-$, 96.9604, 85.0321[M-H-AE-CO_2-H_2O-CO]$^-$	Urine
M22	Apigenin-*O*-glucuronide	$C_{21}H_{18}O_{11}$	12.3	445.0778 (0.3)	269.0449[M-H-GlcUA]$^-$, 175.0241[M-H-AE]$^-$, 113.0240[M-H-AE-CO_2-H_2O]$^-$, 96.9603, 85.0316[M-H-AE-CO_2-H_2O-CO]$^-$	Urine
M23	Apigenin-*O*-sulfate	$C_{15}H_{10}O_8S$	13.4	349.0024 (0.1)	269.0445[M-H-SO_3]$^-$, 117.0350[M-H-SO_3-$C_7H_4O_4$]$^-$, 96.9609	Urine
M24	Eriodictyol [b]	$C_{15}H_{12}O_6$	12.7	287.0558 (-1.2)	225.0700, 201.1115, 175.0711, 151.0041[M-H-$C_8H_8O_2$]$^-$, 135.0456[M-H-$C_7H_4O_4$]$^-$, 107.0130[M-H-$C_8H_8O_2$-CO_2]$^-$	Urine, Feces
M25	Eriodictyol-*O*-glucuronide	$C_{21}H_{20}O_{12}$	10.5	463.0865	383.1953, 287.0555[M-H-GlcUA]$^-$, 175.0246[M-H-EY]-, 151.0033[M-H-	Urine

				(-3.7)	GlcUA-$C_8H_8O_2$]$^-$, 135.0448[M-H-GlcUA-$C_7H_4O_4$]$^-$, 113.0251[M-H-EY-CO_2-H_2O]$^-$, 107.0144[M-H-GlcUA-$C_8H_8O_2$-CO_2]$^-$, 85.0321[M-H-EY-CO_2-H_2O-CO]$^-$	
M26	Eriodictyol-*O*-glucuronide	$C_{21}H_{20}O_{12}$	11.5	463.0884 (0.3)	287.0528[M-H-GlcUA]$^-$, 151.0024[M-H-GlcUA-$C_8H_8O_2$]$^-$, 113.0260[M-H-EY-CO_2-H_2O]$^-$	Urine
M27	Eriodictyol-*O*-sulfate	$C_{15}H_{12}O_9S$	11.2	367.0127 (-0.7)	287.0551[M-H-SO_3]$^-$, 151.0038[M-H-SO_3-$C_8H_8O_2$]$^-$, 135.0419[M-H-SO_3-$C_7H_4O_4$]$^-$, 96.9621	Urine
M28	Eriodictyol-*O*-sulfate	$C_{15}H_{12}O_9S$	12.3	367.0127 (-0.7)	287.0542[M-H-SO_3]$^-$, 151.0023[M-H-SO_3-$C_8H_8O_2$]$^-$, 135.0442[M-H-SO_3-$C_7H_4O_4$]$^-$, 107.0165[M-H-SO_3-$C_8H_8O_2$-CO_2]$^-$	Urine
M29	Eriodictyol-*O*-glucuronide-*O*-sulfate	$C_{21}H_{20}O_{15}S$	9.9	543.0446 (-0.7)	463.0877[M-H-SO_3]$^-$, 367.0121[M-H-GlcUA]$^-$, 287.0553[M-H-SO_3-GlcUA]$^-$, 254.9802, 175.0235[M-H-SO_3-EY]$^-$, 151.0034[M-H-SO_3-GlcUA-$C_8H_8O_2$]$^-$, 135.0455[M-H-SO_3-GlcUA-$C_7H_4O_4$]$^-$, 113.0250[M-H-SO_3-EY-CO_2-H_2O]$^-$, 107.0125[M-H-SO_3-GlcUA-$C_8H_8O_2$-CO_2]$^-$, 95.0149, 85.0310[M-H-SO_3-EY-CO_2-H_2O-CO]$^-$	Urine
M30	Hesperetin [b]	$C_{16}H_{14}O_6$	13.8	301.0720 (0.7)	286.0501[M-H-CH_3]$^-$, 177.0177[M-H-$C_7H_8O_2$]$^-$, 151.0034[M-H-$C_9H_{10}O_2$]$^-$, 134.0372, 107.0132[M-H-$C_9H_{10}O_2$-CO_2]$^-$, 95.0889, 83.0146	Urine
M31	Hesperetin-5-*O*-glucuronide	$C_{22}H_{22}O_{12}$	11.6	477.1034 (-0.9)	301.0716[M-H-GlcUA]$^-$, 286.0470[M-H-GlcUA-CH_3]$^-$, 177.0186[M-H-GlcUA-$C_7H_8O_2$]$^-$, 175.0236[M-H-HE]$^-$, 151.0029[M-H-GlcUA-$C_9H_{10}O_2$]$^-$, 134.0365[M-H-GlcuA-$C_7H_4O_4$-CH_3]$^-$, 113.0243[M-H-HE-CO_2-H_2O]$^-$, 99.0102, 85.0286[M-H-HE-CO_2-H_2O-CO]$^-$	Urine
M32	Hesperetin-7-*O*-glucuronide [b]	$C_{22}H_{22}O_{12}$	11.8	477.1040 (0.3)	301.0719[M-H-GlcUA]$^-$, 286.0477[M-H-GlcUA-CH_3]$^-$, 242.0552[M-H-GlcUA-OCH_2-HCO]$^-$, 175.0240[M-H-HE]$^-$, 151.0026[M-H-GlcUA-$C_9H_{10}O_2$]$^-$, 113.0244[M-H-HE-CO_2-H_2O]$^-$, 95.0146, 85.0315[M-H-HE-CO_2-H_2O-CO]$^-$	Urine
M33	Hesperetin-3'-*O*-glucuronide [b]	$C_{22}H_{22}O_{12}$	12.4	477.1041 (0.6)	360.9595, 301.0702[M-H-GlcUA]$^-$, 286.0452[M-H-GlcUA-CH_3]$^-$, 151.0030[M-H-GlcUA-$C_9H_{10}O_2$]$^-$, 113.0248[M-H-HE-CO_2-H_2O]$^-$	Urine

M34	Hesperetin-*O*-diglucuronide	$C_{28}H_{30}O_{18}$	9.6	653.1362 (0.4)	477.1007[M-H-GlcUA]$^-$, 301.0702[M-H-2GlcUA]$^-$, 175.0221[M-H-HE-GlcUA]$^-$, 151.0018[M-H-2GlcUA-$C_9H_{10}O_2$]$^-$, 113.0238[M-H-HE-GlcUA-CO_2-H_2O]$^-$, 85.0315[M-H-HE-GlcUA-CO_2-H_2O-CO]$^-$	Urine
M35	Hesperetin-3'-*O*-sulfate	$C_{16}H_{14}O_9S$	11.9	381.0282 (-0.9)	301.0706[M-H-SO_3]$^-$, 286.0464[M-H-SO_3-CH_3]$^-$, 205.0829, 165.9884[M-H-SO_3-$C_8H_8O_2$]$^-$, 110.0009	Urine
M36	Hesperetin-7-*O*-sulfate [b]	$C_{16}H_{14}O_9S$	12.3	381.0279 (-1.7)	301.0716[M-H-SO_3]$^-$, 286.0478[M-H-SO_3-CH_3]$^-$, 177.0177[M-H-SO_3-$C_7H_8O_2$]$^-$, 151.0038[M-H-SO_3-$C_9H_{10}O_2$]$^-$, 134.0377[M-H-SO_3-$C_7H_4O_4$-CH_3]$^-$, 107.0136[M-H-SO_3-$C_9H_{10}O_2$-CO_2]$^-$	Urine
M37	Hesperetin-*O*-glucuronide-*O*-sulfate	$C_{22}H_{22}O_{15}S$	9.4	557.0579 (-4.9)	477.1032[M-H-SO_3]$^-$, 383.1930, 381.0252[M-H-GlcUA]$^-$, 301.0719[M-H-GlcUA-SO_3]$^-$, 254.9808, 175.0258[M-H-SO_3-HE]$^-$, 151.0022[M-H-GlcUA-SO_3-$C_9H_{10}O_2$]$^-$, 113.0221[M-H-SO_3-HE-CO_2-H_2O]$^-$	Urine
M38	Hesperetin-*O*-glucuronide-*O*-sulfate	$C_{22}H_{22}O_{15}S$	10.3	557.0597 (-1.8)	477.1038[M-H-SO_3]$^-$, 381.0272[M-H-GlcUA]$^-$, 301.0718[M-H-GlcUA-SO_3]$^-$, 254.9800, 175.0236[M-H-SO_3-HE]$^-$, 151.0035[M-H-GlcUA-SO_3-$C_9H_{10}O_2$]$^-$, 113.0247[M-H-SO_3-HE-CO_2-H_2O]$^-$, 85.0309[M-H-SO_3-HE-CO_2-H_2O-CO]$^-$	Urine
M39	Hesperetin-*O*-glucoside-*O*-sulfate	$C_{22}H_{24}O_{14}S$	9.4	543.0805 (-1.6)	463.1235[M-H-SO_3]$^-$, 383.1926, 301.0705[M-H-SO_3-Glc]$^-$, 287.0521, 151.0010[M-H-SO_3-Glc-$C_9H_{10}O_2$]$^-$, 113.0248	Urine

[a] The losses are: Rha= rhamnose moiety, Glc = glucose moiety, GlcUA = glucuronyl moiety, NE = naringenin, AE=apigenin, EY = eriodictyol, HE = hesperetin.

[b] Confirmation in comparison with authentic standards.

Table S4. UFLC-Q-TOF-MS/MS based identifications of phenolic catabolites in rat urine and feces samples collected 0-48 h after a single oral administration of naringin.

No.	Identified metabolites	Formula	RT (min)	$[M-H]^-$ (Error, ppm)	Fragment ions in negative mode [a]	Source
	Chromone derivatives					
C1	5,7-Dihydroxychromone [b]	$C_9H_6O_4$	9.4	177.0206 (4.9)	133.0272$[M-H-CO_2]^-$, 109.0286, 91.0186, 77.0405	Urine
	Benzenetriol derivatives					
C2	Phloroglucinol [b]	$C_6H_6O_3$	6.7	125.0255 (3.5)	81.0315$[M-H-CO_2]^-$	Urine
C3	Phloroglucinol-*O*-sulfate	$C_6H_6O_6S$	6.7	204.9814 (0.8)	125.0235$[M-H-SO_3]^-$, 81.0360$[M-H-SO_3-CO_2]^-$	Urine
	Phenylpropenoic acid derivatives					
C4	Feruloylglycine	$C_{12}H_{13}NO_5$	9.6	250.0722 (0.2)	232.0602$[M-H-H_2O]^-$, 206.0811$[M-H-CO_2]^-$, 188.0736$[M-H-H_2O-CO_2]^-$, 162.0913$[M-H-CO_2-CH_4-CO]^-$, 132.0309$[M-H-CO_2-C_2H_4NO-CH_4]^-$, 121.0295, 88.0403	Urine
C5	3-(4'-Methoxyphenyl)-2-propenoic acid-3'-*O*-glucuronide	$C_{16}H_{18}O_{10}$	8.3	369.0827 (0.1)	324.0246$[M-H-COOH]^-$, 289.9186, 251.1105, 193.0496[M-H-GlcUA]$^-$, 178.0256$[M-H-GlcUA-CH_3]^-$, 175.0233[M-H-3H4MPEA]$^-$, 149.0590$[M-H-GlcUA-CO_2]^-$, 134.0360$[M-H-GlcUA-CO_2-CH_3]^-$, 113.0228$[M-H-3H4MPEA-CO_2-H_2O]^-$, 85.0293$[M-H-3H4MPEA-CO_2-H_2O-CO]^-$	Urine
C6	3-(4'-Methoxyphenyl)-2-propenoic acid-3'-*O*-sulfate	$C_{10}H_{10}O_7S$	8.8	273.0077 (0.9)	229.0146$[M-H-CO_2]^-$, 193.0496$[M-H-SO_3]^-$, 178.0260$[M-H-SO_3-CH_3]^-$, 149.0597$[M-H-SO_3-CO_2]^-$, 134.0364$[M-H-SO_3-CO_2-CH_3]^-$, 96.9602	Urine
C7	Caffeic acid-4'-*O*-sulfate	$C_9H_8O_7S$	5.2	258.9924 (2.3)	214.9990$[M-H-CO_2]^-$, 187.0061$[M-H-CO_2-CO]^-$, 179.0322$[M-H-SO_3]^-$, 135.0429$[M-H-SO_3-CO_2]^-$, 107.0492$[M-H-SO_3-CO_2-CO]^-$, 93.0352, 80.9649	Urine
C8	Caffeic acid-3'-*O*-sulfate	$C_9H_8O_7S$	5.7	258.9922 (1.7)	215.0004$[M-H-CO_2]^-$, 187.0068$[M-H-CO_2-CO]^-$, 179.0330$[M-H-SO_3]^-$, 135.0446$[M-H-SO_3-CO_2]^-$, 107.0505$[M-H-SO_3-CO_2-CO]^-$, 93.0353	Urine

C9	Caffeic acid-*O*-glucuronide	$C_{15}H_{16}O_{10}$	5.3	355.0665 (-1.5)	283.0792, 179.0340[M-H-GlcUA]$^-$, 175.0272[M-H-CA]$^-$, 113.0257[M-H-CA-CO_2-H_2O]$^-$, 107.0467[M-H-GlcUA-CO_2-CO]$^-$, 85.0315[M-H-CA-CO_2-H_2O-CO]$^-$	Urine
C10	3-(4'-Hydroxyphenyl)-2-propenoic acid	$C_9H_8O_3$	10.9	163.0408 (4.2)	119.0494[M-H-CO_2]$^-$, 93.0356[M-H-CO_2-2CH]$^-$	Urine, Feces
C11	3-(3'-Hydroxyphenyl)-2-propenoic acid	$C_9H_8O_3$	11.5	163.0410 (4.6)	119.0500[M-H-CO_2]$^-$, 93.0325[M-H-CO_2-2CH]$^-$, 91.0572[M-H-CO_2-CO]$^-$	Urine
C12	3-(Phenyl)-2-propenoic acid-4'-*O*-sulfate	$C_9H_8O_6S$	8.6	242.9971 (0.9)	163.0401[M-H-SO_3]$^-$, 119.0509[M-H-SO_3-CO_2]$^-$, 96.9618, 91.0521[M-H-SO_3-CO_2-CO]$^-$	Urine
C13	3-(Phenyl)-2-propenoic acid-3'-*O*-sulfate	$C_9H_8O_6S$	9.4	242.9970 (0.6)	163.0391[M-H-SO_3]$^-$, 119.0498[M-H-SO_3-CO_2]$^-$, 91.0565[M-H-SO_3-CO_2-CO]$^-$	Urine
C14	3-(Phenyl)-2-propenoic acid-4'-*O*-glucuronide	$C_{15}H_{16}O_9$	9.4	339.0724 (0.7)	175.0217[M-H-4HPEA]$^-$, 163.0587[M-H-GlcUA]$^-$, 119.0505[M-H-GlcUA-CO_2]$^-$, 113.0244[M-H-4HPEA-CO_2-H_2O]$^-$, 85.0304[M-H-4HPEA-CO_2-H_2O-CO]$^-$	Urine
C15	3-(Phenyl)-2-propenoic acid-3'-*O*-glucuronide	$C_{15}H_{16}O_9$	10.4	339.0718 (-0.9)	175.0237[M-H-3HPEA]$^-$, 163.0390[M-H-GlcUA]$^-$, 119.0503[M-H-GlcUA-CO_2]$^-$, 113.0247[M-H-3HPEA-CO_2-H_2O]$^-$, 103.0062, 85.0305[M-H-3HPEA-CO_2-H_2O-CO]$^-$	Urine
	Phenylpropionic acid derivatives					
C16	3-(3'-Hydroxy-4'-methoxyphenyl)hydracrylic acid	$C_{10}H_{12}O_5$	11.6	211.0613 (0.6)	193.0496[M-H-H_2O]$^-$, 167.0699[M-H-CO_2]$^-$, 123.0809[M-H-CO_2-C_2H_4O]$^-$, 95.0523[M-H-CO_2-C_2H_4O-CO]$^-$	Urine, Feces
C17	3-(3'-Hydroxy-4'-methoxyphenyl)propionic acid	$C_{10}H_{12}O_4$	12.0	195.0667 (2.1)	160.8426, 151.0739[M-H-CO_2]$^-$, 123.0403[M-H-CO_2-CO]$^-$, 107.0847[M-H-CO_2-CO-CH_4]$^-$	Urine, Feces
C18	3-(4'-Methoxyphenyl)propionic acid-3'-*O*-sulfate	$C_{10}H_{12}O_7S$	8.2	275.0230 (-0.4)	195.0650[M-H-SO_3]$^-$, 177.0547[M-H-SO_3-H_2O]$^-$, 151.0786[M-H-SO_3-CO_2]$^-$, 133.0646[M-H-SO_3-CO_2-H_2O]$^-$, 123.0445[M-H-SO_3-CO_2-CO]$^-$, 81.0360	Urine, Feces
C19	3-(4'-Methoxyphenyl)propionic	$C_{16}H_{20}O_{10}$	10.6	371.0965	327.1336[M-H-CO_2]$^-$, 283.0813[M-H-2CO_2]$^-$, 241.9922, 195.0606[M-H-	Urine

	acid-3'-*O*-glucuronide			(-5.0)	GlcUA]$^-$, 175.0255[M-H-3H4MPPA]$^-$, 151.0760[M-H-GlcUA-CO_2]$^-$, 113.0249[M-H-3H4MPPA-CO_2-H_2O]$^-$, 96.9640, 85.0303[M-H-3H4MPPA-CO_2-H_2O-CO]$^-$	
C20	3-(3',4'-Dihydroxyphenyl)propionic acid	$C_9H_{10}O_4$	7.9	181.0507 (0.3)	163.0384[M-H-H_2O]$^-$, 135.0431[M-H-H_2O-CO]$^-$, 119.0493[M-H-H_2O-CO_2]$^-$, 107.0482[M-H-H_2O-2CO]$^-$, 93.0368	Urine, Feces
C21	3-(3'-Hydroxyphenyl)propionic acid-4'-*O*-sulfate	$C_9H_{10}O_7S$	7.0	261.0074 (-0.3)	217.0180[M-H-CO_2]$^-$, 181.0498[M-H-SO_3]$^-$, 137.0607[M-H-SO_3-CO_2]$^-$, 122.0372[M-H-SO_3-CO_2-CH_3]$^-$, 95.0508, 79.9577	Urine, Feces
C22	3-(4'-Hydroxyphenyl)propionic acid-3'-*O*-sulfate	$C_9H_{10}O_7S$	8.0	261.0077 (0.8)	181.0495[M-H-SO_3]$^-$, 137.0600[M-H-SO_3-CO_2]$^-$, 135.0446[M-H-SO_3-HCOOH]$^-$, 121.0287[M-H-SO_3-CO_2-CH_4]$^-$, 109.0282[M-H-SO_3-CO_2-CO]$^-$, 79.9578	Urine
C23	3-(3'-Hydroxyphenyl)propionic acid-4'-*O*-glucuronide	$C_{15}H_{18}O_{10}$	6.0	357.0825 (-0.5)	181.0486[M-H-GlcUA]$^-$, 175.0261[M-H-3H4HPPA]$^-$, 137.0596[M-H-GlcUA-CO_2]$^-$, 113.0236[M-H-3H4HPPA-CO_2-H_2O]$^-$, 85.0312[M-H-3H4HPPA-CO_2-H_2O-CO]$^-$	Urine, Feces
C24	3-(4'-Hydroxyphenyl)propionic acid-3'-*O*-glucuronide	$C_{15}H_{18}O_{10}$	6.7	357.0825 (-0.7)	181.0491[M-H-GlcUA]$^-$, 175.0258[M-H-3H4HPPA]$^-$, 137.0587[M-H-GlcUA-CO_2]$^-$, 135.0461[M-H-GlcUA-HCOOH]$^-$, 113.0238[M-H-3H4HPPA-CO_2-H_2O]$^-$, 85.0317[M-H-3H4HPPA-CO_2-H_2O-CO]$^-$	Urine, Feces
C25	3-(4'-Hydroxyphenyl)propionic acid [b]	$C_9H_{10}O_3$	10.2	165.0572 (4.2)	121.0634[M-H-CO_2]$^-$, 119.0510[M-H-HCOOH]$^-$, 93.0358[M-H-CO_2-C_2H_4]$^-$	Urine, Feces
C26	3-(3'-Hydroxyphenyl)propionic acid	$C_9H_{10}O_3$	10.8	165.0573 (4.4)	121.0662[M-H-CO_2]$^-$, 119.0507[M-H-HCOOH]$^-$, 106.0429[M-H-CO_2-CH_3]$^-$, 93.0356[M-H-CO_2-C_2H_4]$^-$	Urine, Feces
C27	3-(Phenyl)propionic acid-4'-*O*-sulfate	$C_9H_{10}O_6S$	8.3	245.0139 (5.4)	165.0550[M-H-SO_3]$^-$, 121.0659[M-H-SO_3-CO_2]$^-$, 119.0504[M-H-SO_3-HCOOH]$^-$, 93.0360[M-H-SO_3-CO_2-C_2H_4]$^-$, 79.9596	Urine
C28	3-(Phenyl)propionic acid-3'-*O*-sulfate	$C_9H_{10}O_6S$	8.6	245.0137 (4.7)	165.0548[M-H-SO_3]$^-$, 121.0655[M-H-SO_3-CO_2]$^-$, 119.0503[M-H-SO_3-HCOOH]$^-$, 106.0428[M-H-SO_3-CO_2-CH_3]$^-$, 93.0350[M-H-SO_3-CO_2-C_2H_4]$^-$, 79.9591	Urine
C29	3-(Phenyl)propionic acid-4'-*O*-	$C_{15}H_{18}O_9$	8.2	341.0886	297.1001[M-H-CO_2]$^-$, 279.0890[M-H-CO_2-H_2O]$^-$, 175.0227[M-H-4HPPA]$^-$,	Urine

	glucuronide			(2.2)	165.0544[M-H-GlcUA]$^-$, 113.0240[M-H-4HPPA-CO_2-H_2O]$^-$, 80.9670	
C30	3-(Phenyl)propionic acid-3'-*O*-glucuronide	$C_{15}H_{18}O_9$	8.9	341.0875 (-0.9)	297.0979[M-H-CO_2]$^-$, 165.0553[M-H-GlcUA]$^-$, 121.0661[M-H-GlcUA-CO_2]$^-$, 113.0249[M-H-3HPPA -CO_2-H_2O]$^-$, 80.9662	Urine
	Phenylacetic acid derivatives					
C31	3',4'-Dihydroxyphenylacetic acid	$C_8H_8O_4$	5.1	167.0357 (4.2)	121.0258[M-H-HCOOH]$^-$, 93.0359[M-H-HCOOH-CO]$^-$	Urine
C32/C33	4'-Hydroxyphenylacetic acid or 3'-Hydroxyphenylacetic acid	$C_8H_8O_3$	9.0	151.0408 (5.0)	107.0507[M-H-CO_2]$^-$, 79.9613	Urine, Feces
C34	Phenylacetic acid-4'-*O*-sulfate	$C_8H_8O_6S$	6.0	230.9971 (0.9)	151.0399[M-H-SO_3]$^-$, 121.0484, 107.0497[M-H-SO_3-CO_2]$^-$	Urine
C35	Phenylacetic acid-3'-*O*-sulfate	$C_8H_8O_6S$	7.2	230.9970 (0.6)	187.0068[M-H-CO_2]$^-$, 151.0406[M-H-SO_3]$^-$, 107.0507[M-H-SO_3-CO_2]$^-$, 95.9522, 79.9597	Urine, Feces
C36	Phenylacetic acid-4'-*O*-glucuronide	$C_{14}H_{16}O_9$	6.3	327.0719 (-0.7)	283.0802[M-H-CO_2]$^-$, 175.0207[M-H-4HPAA]$^-$, 151.0384[M-H-GlcUA]$^-$, 113.0228[M-H-4HPAA-CO_2-H_2O]$^-$, 107.0504[M-H-GlcUA-CO_2]$^-$, 99.0078, 87.0091	Urine
C37	Phenylacetic acid-3'-*O*-glucuronide	$C_{14}H_{16}O_9$	7.0	327.0722 (0.2)	283.0808[M-H-CO_2]$^-$, 193.0332, 175.0230[M-H-3HPAA]$^-$, 151.0405[M-H-GlcUA]$^-$,124.0129, 113.0241[M-H-3HPAA-CO_2-H_2O]$^-$, 107.0500[M-H-GlcUA-CO_2]$^-$	Urine
	Benzoic acid derivatives					
C38	3-Hydroxybenzonic acid	$C_7H_6O_3$	6.8	137.0256 (4.9)	93.0339[M-H-CO_2]$^-$	Urine
C39	4-Hydroxybenzonic acid [b]	$C_7H_6O_3$	9.0	137.0257 (5.9)	93.0352[M-H-CO_2]$^-$	Urine, Feces
C40	Benzoic acid-4-*O*-sulfate	$C_7H_6O_6S$	6.4	216.9823 (4.7)	137.0244[M-H-SO_3]$^-$, 93.0356[M-H-SO_3-CO_2]$^-$	Urine
C41	Benzoic acid-3-*O*-sulfate	$C_7H_6O_6S$	6.9	216.9820	137.0241[M-H-SO_3]$^-$, 93.0358[M-H-SO_3-CO_2]$^-$	Urine

				(4.9)		
C42	Benzoic acid-4-*O*-glucuronide	$C_{13}H_{14}O_9$	6.1	313.0565 (0.1)	193.0348[M-H-$C_7H_4O_2$]$^-$, 175.0229[M-H-4HBA]$^-$, 137.0241[M-H-GlcUA]$^-$, 113.0222[M-H-4HBA-CO_2-H_2O]$^-$, 85.0296[M-H-4HBA-CO_2-H_2O-CO]$^-$	Urine
C43	Benzoic acid-3-*O*-glucuronide	$C_{13}H_{14}O_9$	6.8	313.0566 (0.4)	193.0343[M-H-$C_7H_4O_2$]$^-$, 175.0228[M-H-3HBA]$^-$, 137.0241[M-H-GlcUA]$^-$, 113.0246[M-H-4HBA-CO_2-H_2O]$^-$, 93.0381[M-H-GlcUA-CO_2]$^-$	Urine
	Benzoylglycine derivatives					
C44	Hippuric acid [b]	$C_9H_9NO_3$	8.9	178.0522 (6.8)	134.0610[M-H-CO_2]$^-$, 102.0354, 77.0422	Urine, Feces
C45/C46	4’-Hydroxyhippuric acid or 3’-Hydroxyhippuric acid	$C_9H_9NO_4$	7.0	194.0464 (2.8)	150.0550[M-H-CO_2]$^-$, 132.0427[M-H-CO_2-H_2O]$^-$, 100.0045, 93.0357	Urine

[a] The losses are: GlcUA = glucuronyl moiety, 3H4MPEA=3-(3'-hydroxy-4'-methoxyphenyl)-2-propenoic acid, CA=caffeic acid, 4HPEA=3-(4'-hydroxyphenyl)-2-propenoic acid, 3HPEA=3-(3'-hydroxyphenyl)-2-propenoic acid, 3H4MPPA=3-(3'-hydroxy-4'-methoxyphenyl)propionic acid, 3H4HPPA=3-(3',4'-Dihydroxyphenyl)propionic acid, 4HPPA=3-(4'-Hydroxyphenyl)propionic acid, 3HPPA=3-(3'-Hydroxyphenyl)propionic acid, 4HBA=4-hydroxybenzonic acid, 3HBA=3-hydroxybenzonic acid.

[b] Confirmation in comparison with authentic standards.

Table S5. The volumes (mL) of urine collected from 12 aged rats during each time period.

Time (h)	Rat 1	Rat 2	Rat 3	Rat 4	Rat 5	Rat 6	Rat 7	Rat 8	Rat 9	Rat 10	Rat 11	Rat 12
0-4	4.2	5.0	4.2	3.9	2.8	3.2	5.2	3.3	4.4	4.7	6.2	3.0
4-8	2.6	2.1	2.4	1.5	2.5	0.0	0.0	6.4	1.8	0.0	8.9	6.5
8-12	2.3	1.1	3.1	2.2	3.1	3.6	0.0	0.0	2.3	0.0	2.2	8.4
12-24	9.4	8.8	25.5	12.6	14.0	10.9	21.8	6.0	3.3	13.0	6.5	37.5
24-36	3.9	6.0	7.4	4.4	2.8	4.0	5.6	6.4	2.9	0.0	9.5	8.4
36-48	10.5	5.8	13.8	8.0	10.4	9.7	10.6	12.4	6.6	5.9	7.5	23.8

Table S6. The weight (mg) of feces collected from 12 aged rats during each time period.

Time (h)	Rat 1	Rat 2	Rat 3	Rat 4	Rat 5	Rat 6	Rat 7	Rat 8	Rat 9	Rat 10	Rat 11	Rat 12
0-4	685.8	0	0	0	0	0	0	1044.7	0	0	0	0
4-8	0	0	0	377.2	0	0	0	0	0	0	0	0
8-12	0	0	0	0	0	0	0	0	0	0	0	0
12-24	0	0	0	55.9	0	529.2	0	0	1088.1	0	275.1	0
24-36	0	612.7	0	0	0	0	0	1000.3	0	0	208.6	182.0
36-48	739.4	0	0	161.5	527.0	113.8	1073.8	0	0	0	1023.7	0

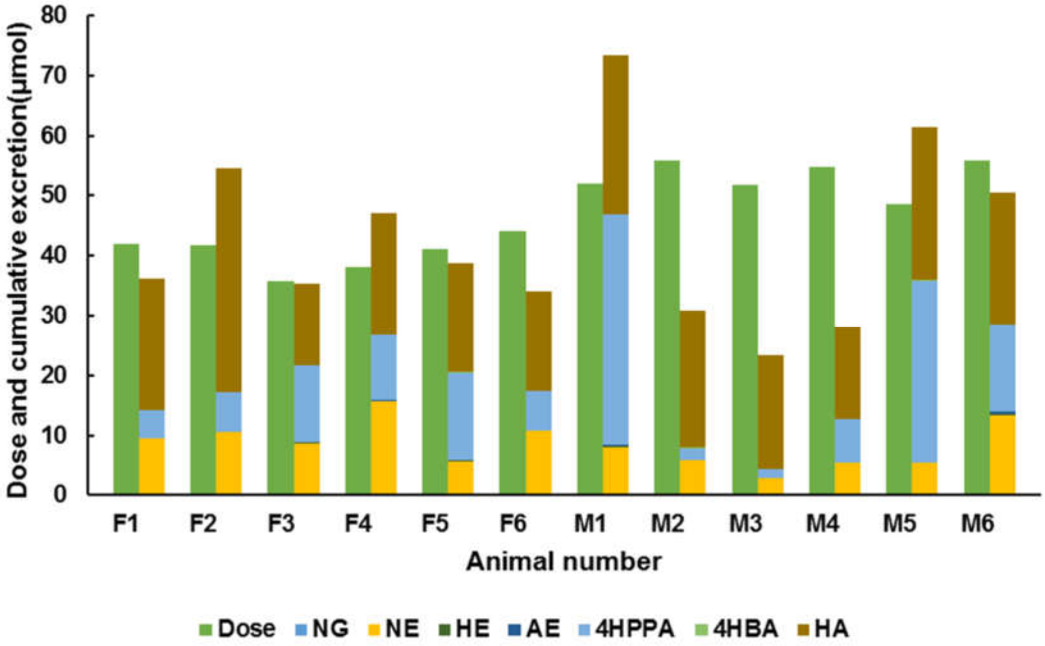

Fig. S1. Dose and cumulative excretion of naringin (NG) and its main metabolites (naringenin (NE), hesperetin (HE), apigenin (AE), hippuric acid (HA), 4-hydroxybenzonic acid (4HBA), and 3-(4'-hydroxyphenyl)propionic acid (4HPPA)) in twelve aged rats.
